# Supplementary material for: COVID-19 healthcare demand projections: Arizona
Source: PLoS One. 2020 Dec 2;15(12):e0242588. doi: 10.1371/journal.pone.0242588 (PMC7710107; doi:10.1371/journal.pone.0242588)
Supplement: Attachment — Submitted filename: Gel_etal-Responses_to_Reviewers.pdf [file pone.0242588.s001.pdf]

"COVID-19 healthcare demand projections: Arizona"
Summary of Changes for the Revision

We thank both reviewers for all of their time and comments that improved the paper significantly. We carefully addressed each one of reviewer 1's comments and concerns, and added a new paragraph in the relevant section to address reviewer 2's suggestion to include more intuition on our results about the initiation of NPIs and their impact. All of the revisions in the new version have been highlighted and marked with tags in the revised version. The following is a summary of the changes we have implemented.

- We have refined the presentation of the model, methodology and results throughout the document.
- We have included more details and intuition on our results and methodology.
- We have explained the novelty of our approach in response to the reviewer comments, and tempering claims to novelty where appropriate.
- We explained the practical use of the different X-factor scenarios that we have simulated in our interactions with the public health officials in the State. We believe that the paper now more clearly explains how scenario based projections can be obtained and used in early stages of an epidemic.

We believe that we were able to address each one of the reviewers' comments. We again thank the editorial team and the reviewers for all their time and attention, and look forward to hearing from you.

Responses to Reviewer 1's Comments

The authors used a compartmental model to describe COVID-19 outbreak in Arizona. Although the overall model structure looks fine, there were some technical issues and inaccuracies. The presentation and organization of the manuscript need further refinement. The simulations were based on very rough assumptions and actual epidemiological relevance is unclear. Substantial revision and/or change of target are recommended for the study to achieve sufficient quality for publication.

Major Comments and Responses:

(R1.C1) *Symptomatic individuals are assumed to remain infectious 6 days after symptom onset, which is not explained in the methods section. This assumption seems inconsistent with the authors' assumption of serial time (serial interval?) of 6 days and lacks empirical evidence (clinically-defined 'recovery' does not necessarily correspond to the loss of infectiousness).*

>>> Thank you for highlighting this interpretation of our assumption and allowing us to clarify in light of some conflation between the period of infectiousness and the period of clinical illness. In our model, we draw on the epidemiology literature that describes the duration of infectiousness for symptomatic individuals that depends on the severity of symptoms and infection. Based on the literature, after symptom onset, mild infections (81% of symptomatic infections) spend an average of 6 days to recovery, whereas the symptomatic individuals with severe and critical infections (19% of symptomatic infections) take much longer to recover and accordingly stay infectious. We assumed that almost all severe infections would be treated in isolation conditions at a hospital (or similar facility) and thus not contributing to community exposure in the same way that mild infections among community-dwelling individuals would be with relevance to our model. We have added a note about our assumption that individuals are no longer infectious after they have recovered (or die).

> "We assume that asymptomatic and symptomatic individuals are no longer infectious after they recover. The duration to recovery or death (i.e., the period of infectiousness) depends on the severity of the disease. "

In addition, we corrected "serial time" to "serial interval" and thank the reviewer for the correction. As serial interval is defined as the time between successive cases in a chain of transmission, in our model this time interval is assumed to correspond to the average amount of time that an individual can be exposed to the virus, become infectious at the end of the incubation period (3 days) and infect someone who will become infectious at the end of 3 days (i.e., a total of 6 days for the serial interval). Based on the literature for COVID-19, we assumed that an individual's case starts at the time that the individual becomes presymptomatic (and infectious) and that individuals are fully infectious during this presymptomatic period, which is modeled to be about 2 days in duration.

(R1.C2) *L221: why is it not 5 + 3 days?*

>>> We thank the reviewer for the opportunity to clarify. Based on the literature for early phases of the COVID-19 pandemic, we accounted for the fact that the average reporting delay (i.e., time between the testing of an infected individual to the reporting of a positive result) was 6 days (we highlighted

this sentence and marked it with R1.C2). Adding 5+3=8 to 6 days gives the 14 day period that we have been using to determine the presumed exposure days.

(R1.C3) *L290: The authors should justify the use of least-square fitting to the number of susceptibles. Fitting to the cumulative data is not recommended as it does not appropriately account for uncertainties in the observed data. Least squares method assumes the data follows a normal distribution with a fixed variance, which may not hold for the epidemic data. The most plausibly, the predicted incidence (considering delay from infection to reporting) should be fitted to the observed case counts (and deaths) using discrete (e.g., Poisson) distributions. Also, most estimates are presented without a measure of uncertainty (confidence intervals).*

>>> We thank the reviewer for the opportunity to clarify this important point about least-square fitting. As the reviewer wisely suggests, indeed we are not using least squares fitting to the number of susceptibles. Rather, we use the actual daily number of reported cases to obtain an X-factor inflated number of individuals that were presumably exposed on the indicated dates (14 days prior to the reporting date). We then use the X-factor inflated number as the data to fit the daily number of exposed individuals indicated by the model. For this purpose, we use the `ParametricNDSolve` routine in Wolfram Mathematica 12.0, which provides a parametric function as the solution to the ordinary differential equations that we have set for the SEIR model. We use this function as a nonlinear model that is a function of the various parameters (such as transmission rate and mortality rate) we can use to obtain the most likely values of the parameters to yield the presumed exposures and deaths data.

That is, for each bin in the model, we obtain a time dependent, function in terms of the model parameters that we are trying to fit. In the case of using the new cases data, the parametric function that expresses the number in the susceptible bin is used to fit to the presumed exposures by considering the daily differences in this function. We have plotted the susceptibles along with the **prediction intervals** for the susceptibles bin, but the fit procedure considers the data on daily presumed exposures and the difference function obtained from the ODE solver as the nonlinear function to fit. We used prediction intervals rather than confidence intervals as the reviewer suggested because a prediction interval is an estimate of an interval in which a future observation will fall with a certain probability. Prediction intervals are typically larger than confidence intervals due to additional sources of variability in the observed value of the response variable of interest (in this case the number exposed daily, or equivalently, change in the susceptibles bin). Given the vast variability inherent in COVID-19 modeling, particularly as it relates to earlier phases of the pandemic, we feel that prediction intervals are the more appropriate interval approach.

The ODE solution of the model provides us with the average values that we expect to see in each bin over time. As such, they are not necessarily discrete values. In our model fits, our goal was not to fit each observation, but rather to obtain parameters that generally explained the behavior over time in an average sense. Due to randomness inherent in pandemic disease spread, we would never expect the data to fit the model exactly. In terms of normality, we incorporated the logic of the Central Limit Theorem to drive our assumption that if we were to simulate this process many times, and calculated the average number in a compartment over time, that average should be normally distributed.

(R1.C4) *Explanation of the model in L170-204 is not well organized. Some epidemiological terms were not appropriately used or defined. Clarity and conciseness are lacking. See also minor comments below.*

As I also find inaccurate uses of epidemiological terms in other parts of the manuscript, I suggest the author seek for proofreading from colleagues with an epidemiology background.

>>> We have implemented corrections in several parts of the manuscript as the reviewer helpfully suggested.

(R1.C5) *Section 1.3: While methodologically being plausible, this initialization method is not novel and is already discussed in a more general model framework of renewal process (for example, see Section 2 of Inaba (2014): https://www.tandfonline.com/doi/abs/10.1080/08898480.2014.891905). Avoid claim of novelty. Moreover, the authors' approach of simply multiplying and shifting the observed incidence neglects uncertainty around both the delays in infection to reporting and underascertainment. Interpolation shown in Fig. 6 is obviously overfitted. The authors should account for these uncertainties that might have affected their results, especially the confidence intervals.*

>>> We appreciate the opportunity to clarify that the novelty of our approach is in its application to early phases of an outbreak or pandemic when reliable and timely data are not available.

We also appreciate the opportunity to clarify that the interpolation in Figure 6, by definition, is an interpolation, and does not attempt to represent a fit. To that end, we respectfully disagree with the reviewer's assertion that the interpolation is overfitted. Rather, we have taken the approach of using functions of arbitrary degree to obtain interpolated functions that pass through any number of data points. We simply used the interpolation to compute the convolution to estimate the number in each compartment at a desired point in time (i.e., the date we would like to initialize). Note that we keep the transmission rate to zero and only use the presumed exposures to do this. Hence it was important to not introduce any additional errors to the presumed exposures through the interpolation. Alternatively, we could have discretized time and calculated the estimated number in each bin after t days of time, given the presumed exposures in each day. For example, the process of estimating the number in the symptomatics compartment on day t would involve taking the presumed exposures for each day, calculating the expected number that will be in the symptomatic compartment by time t out of those presumed exposures, and summing over all of the days prior to date t. The convolution essentially performs this calculation on a continuous time basis, and nothing else. To make this point clear, we added the following into the manuscript:

> "Alternatively, we could have discretized time and calculated the estimated number in each bin at a desired time, given the presumed exposures in each day prior to that point. For example, the process of estimating the number in the symptomatic compartment on a given day t would involve taking the presumed exposures in each day prior to that date, calculating the expected number that will be in the symptomatic compartment by day t out of those presumed exposures, and summing over all of the days prior to date t. The convolution essentially performs this calculation on a continuous time basis."

We are intentionally not incorporating any additional variability into the process because what we would like to see is the compartment composition if the presumed exposures obtained by X-factoring the data were the only source of exposures in the system.

We were not completely certain which part of the reference the reviewer was referring to in the comment, and we could not ascertain how the indicated reference was relevant to the method we were using to

initialize the model. This lack of clarity was helpful to us to indicate the opportunity for us to further clarify our methods to enhance the reader's understanding and so we thank the reviewer for uncovering the unintentional ambiguity.

With regard to the reviewer's comment on novelty, we appreciate the opportunity to clarify that the novelty of our approach is in its application in the early phases of an outbreak condition prior to the broad availability of reliable epidemiological data. As our X-factoring scheme was based largely on the observations from novel coronavirus and the testing/detection status in Arizona in 2020 in the absence of another established modeling paradigm for the early outbreak condition, we thank the reviewer for sharing the Inaba 2014 reference, and respectfully disagree with the assertion that our utilization approach has already been described in that paper.

(R1.C6) *Discussion on the ICU cases, mortality rate, and the reported number of deaths is weak in logic. Given the number of confirmed cases and death, the rate of underreporting ("X factor") and the mortality rate are two completely correlated parameters. The best approach is to fit the model simultaneously to case and death data. If 1.67X scenario does not match the observed number of deaths as L344-345 suggests, this means either the assumption of* $X = 1.67$ *or other model settings may be wrong. It did not make sense to me that the authors stick to 1.67X scenario when the model output is inconsistent (mortality ¿ 100%).*

>>> We thank the reviewer for the opportunity to clarify the challenges in the ways that epidemiological data in the early days of an outbreak are reported via different bureaucratic mechanisms and with a variety of time delays due to factors including supply chain deficits, database management challenges, staffing shortages, and adjudication reporting delays particularly related to investigated death cases as well as the intrinsic lag in deaths as an outbreak indicator. The mortality rate, when defined in the general sense, is definitely correlated to the rate of underreporting. However, we note that the mortality rate that we are fitting with this approach is the rate of individuals with critical infections, most likely in the ICU departments of Arizona hospitals, whereas the case rates exist throughout the broad community. That presumed localization to the ICU setting for the majority of death cases as opposed to no similar localization for cases write large provides the rationale driving our decision to set these (i.e. case and death) parameters through separate model fits. It is however, worthwhile to note that we conduct these fits iteratively, to reach a general consensus between the two fitting procedures, one using the new cases data, and the other one using the deaths data.

In the event that the reviewer is interested in the real-world experience behind the X=1.67 projections, we are glad to share that 1.67X projections were used as a lower bound projection, and was ratified by the state's epidemiology division leadership in our discussions with the Arizona Department of Health Services. We note that this case of X=1.67 represents a highly optimistic case where ALL of the symptomatic individuals were detected. As such, it was important for us to demonstrate that this case and hence this assumption falls significantly short of explaining the deaths observed in Arizona over time. This optimistic projection approach allowed us to motivate higher values for the X-factor. Over time, as more and more data became available, we were able to see that indeed our projections using an X-factor of 4 were significantly more representative of the epidemic in Arizona. Hence, we respectfully disagree with the reviewer's comment on this and emphasize the importance of the real-world use of the 1.67X case with the following paragraph in the manuscript.

"For the 1.67X scenario, this resulted in a fitted υ value of 1.23, meaning that the 1.67X scenario did not generate sufficient number of patients in the ICU to explain the reported deaths in Arizona. Hence, we used an υ value of 0.99 for the 1.67X runs. This result shows that 1.67X scenario, which basically represents a case where all of the symptomatic individuals were detected by the testing effort, was overly optimistic and fell severely short of explaining the observed deaths in Arizona over time. However, this case still serves a significant practical purpose, since it demonstrates that the actual X-factor in the system is higher than 1.67, and gives a lower bound on the projections that one may develop based on the available data. In fact, this case was highly useful in our conversations with the public health experts in the State, to guide their testing efforts and reflect a reasonable picture of the epidemic during the early stages. "

(R1.C7) *Section 2.1: No citation on the possible "summer effects" on the transmission of SARS-CoV-2? If the authors wish to discuss temperature effects, sufficient background needs to be provided to the readers. Currently, the arbitrary choice of 3 scenarios are not of epidemiological interest since the actual relevance is unknown.* >>> We thank the reviewer for providing the opportunity to further discuss the rampant speculation about a so-called summer effect that drove policy decision making in the real-world setting early in the COVID-19 pandemic. Indeed, we explicitly discuss the fact that at the time of our study, representing early experience in the outbreak, there was no consensus on the so-called summer effect. The scenarios, as we indicate in the manuscript, are purely generated to demonstrate the impact of a favorable summer effect on the hospital projections in Arizona. For example, given that the Arizona hospital capacity is around 8000 inpatient beds the summer effect scenarios allowed the public health policy makers to appreciate the likelihood of a given summer effect and the resulting impact of it. This exercise was useful in our conversations with the public health officials in the State. To wit, by providing projections related to a possible so-called summer effect early in the pandemic, we created the context by which our projections were able to disprove the existence or impact of such an effect, thus motivating policy makers to enact more aggressive non-pharmaceutical interventions. To explain this point, we added the following to the manuscript.

"In particular, we have simulated four scenarios with no summer effect, 25% decrease in transmission rate on 5/29, 25% transmission rate on 6/12, and a 50% decrease on 6/12 and calculated the projected hospitalizations in each case. The projections under these cases were useful for public health officials in the State to appreciate the fact that given all the debate with respect to a favorable summer effect, it was likely that hospital resources in Arizona during the summer of 2020 will be significantly constrained. These projections are provided here to demonstrate how we used such scenarios to communicate these issues, rather than an expectation that these scenarios are epidemiologically relevant or supported by data or studies on potential summer effects."

(R1.C8) *Section 2.2: I am not sure how useful the "reinitiated NPI" scenarios are when the assumed reduction in transmission cannot contain the epidemic ($R_{eff} > 1$). Given that the previous state-home-order lasted only for 1.5 months (lifted even before the CDC criteria were met according to Introduction), scenarios assuming the same level of restriction continued for at least 3-6 months (or much longer if continued until the epidemic is over) may not be realistic.*

>>> Again, our goal in generating these scenarios was primarily to demonstrate useful cases for public health officials to appreciate. We believe that we make this point in the section, and emphasize that these projections were generated to help guide decisions to intensify hospital resources, ventilators, etc.

Minor comments and responses:

(R1.C9) *Figures 1 and 2: It would be helpful if the daily numbers are also shown.*

>>> We thank the reviewer for this suggestion. The figures were provided to help the reader visualize the data that we used to fit the models and obtain the projections. We have provided a link to the source of the data; interested readers can observe the daily numbers from the Arizona Department of Health Services data dashboard at

`https://www.azdhs.gov/preparedness/epidemiology-disease-control/infectious-disease-epidemiology/covid-19/dashboards/`.

ADHS provides a number of useful visualizations of the data at this web page that exceed the scope of the paper but may be interesting to readers and relevant to other analyses.

(R1.C10) *L102: time from infection to exhibiting infectiousness is called the latent period, not incubation period.*

>>> We thank the reviewer for this correction, which we have implemented in the paper.

(R1.C11) *L168: Equations/through*

>>> Done.

(R1.C12) *L169: The definition of the force of infection is the rate of new infection per susceptible population, which should be $-dS/dt/S = \beta_t\lambda(t)$ in this model.*

>>> We thank the reviewer for this helpful correction, which we have implemented in the paper.

(R1.C13) *L174-175: It is vague what "these compartments" points to. Also, "patients" usually refers to people visiting hospitals and does not fit in context (presymptomatic individuals are not really patients; "symptomatic patients" suggests they have medically-attended).*

>>> We thank the reviewer for the opportunity to clarify the language in this section. We have changed that part of the noted sentence to read as follows:

> "COVID patients with severe or critical infections who are typically receiving care in the hospital (i.e., compartments $\mathcal{H}$, $\mathcal{C}$ and $\mathcal{B}$) are relatively well isolated via institutional infection control measures."

(R1.C14) *L177-178: the rate at which susceptible individuals get exposed to the infection is $\beta_t\lambda(t)$, not β_t. This sentence is unclear if it means the product of β_t, $\lambda(t)$, and $S(t)$ the authors should rearrange the whole sentence for better readability. Also, the word "exposed to the virus" is vague and should be avoided in this context. "Infectious contact" itself is usually regarded as exposure in epidemiology, whether or not such contact results in infection. Getting into "Exposed" compartment should be termed differently (e.g., become infected).*

>>> We agree with the reviewer's helpful suggestion, and so we removed the sentences in question and reorganized the paragraph to make the terms in the ODEs more clear.

(R1.C15) *L179-180: not clear what is meant to say: "weighted proportion of infectious individuals accounting for the reduced infectiousness of asymptomatic and hospitalized individuals" or something?*

>>> We thank the reviewer for this helpful suggestion. We have removed sentences and reorganized the paragraph to clarify the transmission terms in the ODEs.

(R1.C16) *L181-182: this definition of transmission rate is incorrect. As $\lambda(t)$ represent proportion infectious, β_t should be regarded as contact rates x transmission probability, regardless of whether the contactee is infectious or not.*

>>> We thank the reviewer for this helpful clarification and agree with the comment. The first sentence in the following paragraph states exactly the definition that the reviewer is citing. In addition, we have modified the paragraph to clarify this point.

(R1.C17) *L191-199: this explanation is redundant and nothing more than rephrasing that β_t is a product of contact rate and transmission probability given contact. Figure 4 also seems trivial unless empirical data on the number of contacts in the study population is given. Also, it does not make sense to me that doubling the number of contacts from 10 to 20 does not result in halving the probability of transmission, which instead becomes 0.013 (not 0.01??).*

>>> We thank the reviewer for this helpful critique in the context of this paper. Figure 4 was a visual aid that we used to communicate the concept with public health officials, and was found to be useful to explain the impact of various NPIs at a high level. However, in response to the reviewer's note, we have removed the figure and the corresponding explanations from this manuscript. The deletion is marked as R1.C17. We modified the paragraph to make the points we intended to make with respect to the measures to reduce the transmission rate via reducing the rate of contacts versus reducing the probability of transmission given contact with an infectious individual.

(R1.C18) *L262 and thereafter: the word "bin" is misleading. It is "compartment", isn't it?*

>>> We appreciate the clarifying comment and have replaced all instances of the word "bin" with the word "compartment".

(R1.C19) *L268: the term "loading scenario" never explained*

>>> We thank the reviewer for illuminating this important oversight. We have attended to this omission, and now define what we mean by loading scenario more clearly in the paragraph (highlighted and marked with R1.C19).

(R1.C20) *Figure 10: Cumulative curves are not suitable to show the model fit as they tend to look better than it actually is. Use the daily number of deaths instead.*

>>> We thank the reviewer for this suggestion, and the opportunity to clarify the nature of the real-world data on which our work is constructed. Due to the way that deaths are reported and used by public health officials and policy makers in Arizona, we have found the cumulative curves approach to be a more useful way of presenting our results to policy makers. It is not our goal to predict daily death values, as this is quite pointless and probably impossible to do with sufficient accuracy due to the plethora of sources of uncertainty involved in a complex process like the extant public health emergency. However, the prevailing data focus in the local public health and policy making community

has prioritized the display of cumulative data curves. The public health agencies that we have been working with have, therefore, been able to make meaningful use of the cumulative projections that we have been able to provide with satisfactory accuracy.

(R1.C21) *Section 2.2: The use of the term "NPI" here is inappropriate. NPIs do not only refer to lockdowns or stay-home-orders.*

>>> We thank the reviewer for the opportunity to revisit our use of the term NPI. We reread the section and the use of the term "NPI" in the section. We do not feel that our usage of the term implies that NPIs only refer to lockdowns or stay-at-home orders. We intentionally used the term in a vague sense, since for example, mask usage in Arizona after June 20 has resulted in reduction in transmission rate similar to the levels described in this manuscript. We do not feel that our use of NPI in Section 2.2 implies the narrow usage that the reviewer suggests, and respectfully disagree with the reviewer's interpretation of our intention.

(R1.C22) *L420: why is 1.67X "conservative"?*

>>> We clarified this point further in the manuscript, and invite the reviewer to refer to our response to R1.C6 above.

(R1.C23) *L490: "proposed a methodology" is an overstatement as this is an application of an existing model to the Arizona data.*

>>> We thank the reviewer for the opportunity to reflect on this interpretation. We respectfully disagree that this is an application of an "existing" model to Arizona data. Certainly, the use of a carefully parameterized SEIRD model is not a novel approach, and perhaps that is the reviewer's point. However, our use of backcasting techniques as well as transmission rate fits was a novel approach in the context of early phases of a novel viral pandemic without extensive data available. Therefore, we are confident in our assertion of novelty in the extant context, particularly as it has not been described elsewhere.

(R1.C24) *L510: "biologically plausible" Which parameter, and how? I do not see that many biological papers in the reference list.*

>>> We thank the reviewer for this critique, as it correctly points to our internal inference of biological plausibility based on our group's subject matter expertise related to virology and human physiology, rather than peer-reviewed biological literature. We have removed the word "biological" from this section of the paper.

(R1.C25) *L524: I do not fully agree that the model is "locally contextualized" just because the model is fitted to the local data. The model structure is not locally specific and most parameters are cited from the literature.*

>>> We thank the reviewer for this helpful critique. We removed the term "locally contextualized".

Responses to Reviewer 2's Comments

The paper presents a mathematical model to predict the impacts that the COVID-19 epidemic in Arizona will have on the state's healthcare sector. The topic is interesting and worth of investigation. The paper is well organized and the results meaningful.

A suggestion to help improve the paper are as follows:

(R2.C1) *I suggest describing the general trend shown in Tables 2, 3, and 4 in greater detail to supplement the current paper's comparison of the varying predictions given different NPI restoration dates.*

>>> We thank the reviewer for this helpful suggestion. We added a paragraph to the discussion section to discuss the trends, as suggested.

> "Further examination of the trends associated with the timing of NPI initiation indicates that the most significant reductions following the successful initiation of an effective NPI is realized within the first two months of policy change. This observation reflects the nature of infection spread in the community for a viral illness with exponential growth potential, as observed with COVID-19. Just as exponential growth experiences a long initiation period in a population prior to sharp rises in case counts, exponential decline in case counts occurs relatively quickly in the population when the transmission routes are attenuated by NPIs, followed by a longer period of more gradual decline that reflects the gradual onset period of the epidemiological curve. It is important to note that the projected reductions in total infections, hospitalizations, and deaths with NPI initiation presume widespread community uptake of any intervention. Poor public adherence to NPI intervention policies are unlikely to result in significant attenuation of viral spread, as policy alone without broad public action is powerless in a pandemic."
