# Supplementary material for: COVID-19 healthcare demand projections: Arizona
Source: PLoS One. 2020 Dec 2;15(12):e0242588. doi: 10.1371/journal.pone.0242588 (PMC7710107; doi:10.1371/journal.pone.0242588)
Supplement: Attachment — Submitted filename: Response to Reviewers.pdf [file pone.0242588.s002.pdf]

"COVID-19 healthcare demand projections: Arizona" Summary of Changes for the Revision #2

We thank the review team for their comments on the first revision of our paper. Revision#1 sufficiently addressed Reviewer 2's comments. Below, we provide responses to Reviewer 1's comments on Revision 1. We believe that we were able to clarify all of the remaining issues from Revision 1. In particular, we have rewritten the justification of various parameters used in the model and clarified several aspects of our methodology. All of the changes are highlighted in the new version of the manuscript, and marked with the corresponding comment number.

Responses to Reviewer 1's Comments on Revision 1

I appreciate the authors' effort to improve the manuscript for better clarity. The revision did address many of my concerns, while I have a few more comments as bellow on the responses of the authors which I do not fully agree with/believe requires more clarification. Especially, the inconsistent definition of time interval in the model may be a critical point that can change the conclusion of the study.

R1C1-duration of infectiousness: I still don't know which literature the authors referred to to obtain duration of infectiousness. Ref [6] and [22] are shown in Table 1, but [6] is a modelling study using 6.5 days as generation time (without citing data) and I couldn't find any mention on the duration of infectiousness in [22]. I would appreciate if the authors could specify which part of the references they cited the duration from.

R1C1-serial interval: It still seems to me that the authors' explanation that the serial interval is given as 3 days incubation period of a primary case + 3 days incubation period of a secondary case is incorrect. First of all, incubation period (time from infection to "symptom onset") in this model is not 3 days but 5 days. Even if the authors intend to refer to the latent period (then it will be generation time instead of serial interval), infection at the end of 3 days of the latent period will not correspond to the "mean" serial interval because infection can happen between the beginning and the end of infectious period (hence 3+3 is the shortest possible serial interval). Exact computation of generation time in this model is not straightforward because of the combination of Ip and Is compartment with different infectiousness (but could be done by constructing the survival function of infectiousness: multicompartment-equivalent of Eq 6 in Inaba 2014), but it is at least 3 (latent) + 6 (mean duration of Is) + (duration of Ip modified by lower infectiousness). I believe that the mean serial interval being longer than 3 days could also be checked by simulating this model with explicit tracking of generations. This inconsistency in the model setting should be addressed.

>>> Thank you for allowing us to clarify this point. We have rewritten that entire section to explain our logic and sources in estimating the parameters related to latent period and the serial interval. We also explained how we arrived at an average duration of infectiousness, given the multicompartment nature of our model. We explained our approximation to arrive at an average duration of infectiousness. We then showed more explicitly how this estimate along with the serial interval estimates from the literature was used to arrive at an average latent period of 3 days and presymptomatic period of 2 days. Below, we include the new section from the paper. Some parts of the edited paragraphs could not be highlighted but the changes involve all paragraphs between lines 101 thru 141. We have also modified Table 1 where we indicate the sources for the parameters used in the model.

> Our model defines separate compartments for asymptomatic and presymptomatic individuals to explicitly account for differing rates of transmission and differing durations of illness. Individuals

who are exposed to the virus go through a latent period (modeled by a rate of ζ) during which they are exposed but not yet infectious. Following this latent period, we assume that some infections will recover without developing any symptoms (i.e., $\mathcal{I}_{\mathrm{a}}$). The remaining infections will be presymptomatic for a period of time (modeled by a rate of δ) meaning that their symptoms will remain subclinical, but they are assumed to be infectious during this time (He et al. 2020). For symptomatic infections, the incubation time is taken to be 5 days, based on estimates by (Park et al. 2020; Lauer et al. 2020; Linton et al. 2020; Zhang et al. 2020; Li et al. 2020). Therefore, for the novel coronavirus with variation in symptom onset, we use an averaged infectious period to estimate the latent period from estimates of serial interval. Recall that an approximation for the average latent period can be obtained by subtracting half the average infectious period from the average serial interval. Before explaining our assumption on the duration of infectiousness, we note that we used 6.6 days as the serial interval, which was supported by the early estimates available in the literature at the time of our study (Ki 2020; Li et al. 2020; Bi et al. 2020; Park et al. 2020) and was relevant to the local context that we were trying to model. Subsequent estimates of serial interval were slightly lower (around 5.5 days) due to faster identification of cases and contact tracing efforts (Zhang et al. 2020; Tindale et al. 2020; Du et al. 2020; Nishiura et al. 2020; Rai et al. 2020).

We assume that asymptomatic and symptomatic individuals are no longer infectious after they recover. The duration to recovery or death (i.e., the period of infectiousness) depends on the severity of the disease. We modeled a number of observed variations on how symptomatic individuals experience COVID-19. After the presymptomatic period, a large fraction, 81% (denoted by ρ in the model) estimated by (Wu and McGoogan 2020; Zhou et al. 2020), of symptomatic individuals go through a 6-day infectiousness period with relatively mild symptoms that do not require hospitalization. Similarly, we assumed that asymptomatic patients recover at a rate of $\gamma = 1/6$, corresponding to an average recovery duration of 6 days after the preinfectious period. This assumption was based on the estimates of infectious periods for mild infections available at the time of the study, which was somewhat scarce, since most of the available data was on severe or critical patients getting care at the hospital. We used (Wolfel et al. 2020) to justify an "average" duration of infectiousness of around 7 days for these patients, which subsequently proved to be a good assumption as shown by (Tenforde et al. 2020). The remaining 19% of symptomatic patients develop a severe or critical infection and seek care at a hospital. We assume that these patients are generally isolated and infect others at a much reduced rate. We discuss this assumption further below. Using an average duration of infectiousness calculated as 0.4*6 + 0.6*8=7.2 days (using a weighted average of durations of infectiousness for asymptomatic and symptomatic patients), we can approximate average latent period as 6.6-3.6=3.0 days. Given the 5 day estimate for the incubation period, this gives us 2 days for the presymptomatic duration (modeled with rate δ).

R1C1 "Based on the literature for COVID-19, we assumed that an individual's case starts at the time that the individual becomes presymptomatic (and infectious) and that individuals are fully infectious during this presymptomatic period, which is modeled to be about 2 days in duration.": I'm not sure what "individual's case" refers to, but serial interval is specifically defined as time between symptom onsets of linked cases, in which sense the transition from Ip to Is should be regarded as an onset. Also, "individuals are fully

infectious during the presymptomatic period" sounds inconsistent with the model assumption that those in IA compartment has infectiousness 0.55 times that in Is, according to the definition of λ.

>>> We thank the reviewer for this observation, and note that the reviewer is correct about the IP to IS transition corresponding to symptom onset. We do not believe that it is inconsistent to assume that presymptomatic individuals, who will later on develop symptoms, are in fact contagious during this short period of time. In fact, these presymptomatic individuals (probably due to the fact that they are not yet showing symptoms but have a significant viral load) were observed to be as infectious as symptomatic individuals in studies. A good example of this body of evidence is the results presented by He et al. (2020) in Nature Medicine, who state "...We observed the highest viral load in throat swabs at the time of symptom onset, and inferred that infectiousness peaked on or before symptom onset." This point of view reflected the understanding at the time of the writing of this manuscript. This reference was already cited in our paper. We added this reference in addition to the other paper already referenced in this section to clarify this point.

R1C3-fitting target: If the least-squares fitting was used for the daily counts of cases, I believe the description between L292-298 should be rewritten because it is misleading. It is not indicated that the daily count was used; moreover, showing the number of susceptibles in the context of fitting implies that it might have been the target of fitting.

>>> We thank the reviewer for the opportunity to clarify that the least squares fitting was not used for the daily counts of cases. The daily counts of cases were processed to obtain what we call "presumed exposures" by multiplying them by the X-factor that we assume, and obtaining a likely exposure date by subtracting 14 days from the reporting date of a case. The number of susceptibles at a given date is then equal to the initial number of susceptibles in the population minus the cumulative number of exposed individuals. We modified the section as follows to clarify this point, highlighted and marked it as R1.C3.

> ...We then use the X-factored presumed exposures to obtain the corresponding number of susceptibles, which is equal to the initial number of susceptibles in the population minus the cumulative number of exposed individuals, and use a nonlinear model fit procedure to estimate $\hat{\beta}_1$, $\hat{\beta}_2$ and $\hat{\beta}_3$. ...

R1C3-discrete distribution: I understand that the ODE solution gives noninteger values, but this does not prohibit the use of discrete distributions such as Poisson distribution (mean of Poissson distribution can be noninteger). The central limit theorem assures approximate normality if the process is simulated many times as the authors suggest in the response, but because the available data is only one realization of the process and not the average of many simulations, this does not necessarily apply. Instead, the central limit theorem may ensure that the Poisson model is approximately normal (if the mean is large enough), but then the least-squares relative error method should be used instead of absolute error (because Poisson(μ) $\approx$ Norm(μ, μ)).

>>> We admit some challenge in fully understanding what the reviewer is asking for in this comment, as we do not report residuals or error values directly in the paper. Therefore, we are uncertain about the source of the confusion. We assume that the reviewer is referring to the "Least Squares relative error regression" type approaches where the objective function to be minimized is the error divided by the observation value, so that the relative size of the errors are considered in the fitting operation. In cases where the observations may have different magnitude, it does make sense to scale the errors with the magnitude of the observations and use relative errors. In our case, we try to find the transmission rate that would, on average, give us the presumed

exposures that we obtain from the daily case counts over time. Since the values of the "observations" (more accurately, the presumed exposures that we infer from the daily case counts data) are relatively close to each other, we do not see the point of using relative errors in this case, and thus do not report error values.

We would like to reiterate that the model that we are fitting is a nonlinear model. As such, the optimization procedure to find the best model parameters requires numerical methods (since no analytical solutions as in the case of a linear fit is available). Hence, we decided to use a well established library from Wolfram Mathematica to do the fitting, and used the `NonlinearModelFit` function. Some more detailed information about the library can be found in https://reference.wolfram.com/applications/eda/FittingDataToNonlinearModels.html. The method uses sum of squared errors, not absolute errors. The nonlinear model form that we feed Mathematica for the model fitting is what we obtain from the parametric solution of the ODE, obtained by Mathematica's `ParametricNDSolve` library.

We appreciate the opportunity to reiterate that this part of our analysis is relatively standard and is used commonly in efforts to fit ODE models using whatever actual data is available (in our case, the daily case counts during that period of time). We are not making any assumptions on the distribution of any random variable or process as the reviewer is suggesting. All we do here is to find the model parameters (transmission rate) such that the ODE model's solution (which is unique for a given initialization of the model) corresponds to the general behavior that we observe through the case counts data over time.

R1C5-reference to Inaba 2014: Apologies for my comment not specific enough, I wanted to especially highlight Eq 5 in Section 2, which shows exactly what is done as initialization in the current paper. Note that the model here is a generalization of compartmental models and includes the SEIRD model as a special case. The second line of Eq 5 represents the "backcasting" of the past states of compartments ($y(t, \tau, \xi)$ in a general form), while the first line predicts the onward transmission.

>>> With apologies to the reviewer, we are still not understanding the point that the reviewer is making with this comment. We appreciate the opportunity to reiterate that the only claim of novelty we make in the entire Section 1.3 is the following sentence, related to using an X-factoring scheme to obtain the likely number of infections from the given number of confirmed daily cases. That sentence reads:

> ...To account for the large rate of undetected infections, we have devised a novel approach using an X-factor initialization scheme where we multiply the number of eventually detected-exposed individuals by the X-factor to obtain the underlying overall exposures on a given presumed exposure day...."

Our claim of novelty is only in reference to the X-factoring, or inflating the case counts to account for undetected cases. We appreciated the opportunity introduced by the reviewer's comment to carefully review our manuscript and reaffirm that we do not make any claims that we were the first ones to make this calculation. We are simply explaining the process that we used to obtain the results given in the paper.

As far as we can see, Inaba (2014) has no mention of using real life data to initialize a model. From the abstract of the paper, "...The main result is that the positive solution of the final size equation gives the lowerbound of the intensity of any epidemic starting from a host population composed of susceptible and a few infected individuals who spread on a noncompact domain of the trait variable."

In our case, we are using observed case counts from real world epidemiology data, inflating them with an X-factor, and inferring a likely exposure date, and then using the f_i functions, calculating a total average number to expect in each compartment at a given desired date of initialization by summing over all of the

presumed exposures. We do not indicate the use of the f_i functions as novel, nor do we think that it is significant enough to claim novelty, since we obtain it numerically as a parametric function by simulating the ODE with a single infection at time zero, in a context with no transmission. This technique allows us to observe the average number (i.e., fraction) we expect to see in each compartment over time. We acknowledge that our approach probably corresponds to the expressions in the equations (4), (5) or (6) in Inaba (2014). However, what we are doing here is so basic a mathematical operation that we fail to see the relevance of the reference. We reiterate that we claim no novelty with respect to theoretical results. Rather, we assert that our context of using real life data in the early phases of an emerging pandemic is completely different than the theoretical case that is studied in Inaba (2014). We contemplated the possibility of citing the paper as the reviewer suggests, but we are concerned that an intellectually honest citation of Inaba (2014) would require us to also cite a very large number of additional papers that are of similar distance/relevance to the current article, and would not yield any meaningful contribution to the meaning or understanding of our manuscript.

R1C7-Background of summer effects: I understand that the evidence at the earlier time was mixed and the authors may have had to draw scenario projections based on rough assumptions. Still, I believe it would be useful to provide the readers with context by clarifying what data on summer effects was available at the time of study (I believe at least some preliminary data or reasoning existed that hypothesized the summer effects), and how our understanding has changed since then.

>>> We thank the reviewer for this comment and the opportunity to clarify our manuscript regarding the summer effect discussion. We have modified the relevant section as follows:

> At the time of the writing of this manuscript, there was significant debate about the impact of higher summer temperatures in large regions of Arizona on the transmission rate of COVID-19; there was no consensus in the peer-reviewed literature. This conversation about possible temperature effects was important since some local policymakers were raising the possibility that a heat-mediated summertime transmission decline could justify lifting NPI policies implemented during the relatively cooler spring months. A so-called summer effect would include a potentially suppressive effect on virus survivability in the extremely elevated temperatures and UV radiation of the desert Southwest. Simultaneously, the extreme summer heat in Arizona's population centers also creates a behavioral effect, changing patterns of indoor and outdoor activity in Arizona's desert environment. In essence, Arizona's summer effect behaviorally mimics the winter effect in more temperate regions, as people seek heat relief in indoor environments. Baker et al.'s Baker et al. (2020) published in May 2020 indicates that nonpharmaceutical control measures may moderate the pandemic-climate interaction through susceptible depletion. In other words, human behavior can dominate any climate effect at this early stage of the pandemic, which clearly deviates from the behavior observed for endemic infections.
>
> Our experience in Arizona confirms the findings in this paper; the transmission rate observed from data was more shaped by the NPIs in effect than by the weather. At the timing of this study, however, given the uncertainty about a potential summer effect, particularly on virus survivability, we found the following analysis to useful to demonstrate the sensitivity of outbreak dynamics to the transmission rate β when presenting our results to public health officials. The scenarios demonstrate the impact of different levels of favorable summer effects on projected hospitalizations.

References

Baker, R. E., Yang, W., Vecchi, G. A., Metcalf, C. J. E., and Grenfell, B. T. (2020). Susceptible supply limits the role of climate in the early SARS-CoV-2 pandemic. *Science*.

Bi, Q., Wu, Y., Mei, S., Ye, C., Zou, X., Zhang, Z., Liu, X., Wei, L., Truelove, S. A., Zhang, T., et al. (2020). Epidemiology and transmission of COVID-19 in Shenzhen, China: Analysis of 391 cases and 1,286 of their close contacts. *The Lancet Infectious Diseases*.

Du, Z., Xu, X., Wu, Y., Wang, L., Cowling, B. J., and Meyers, L. A. (2020). Serial interval of COVID-19 among publicly reported confirmed cases. *Emerging infectious diseases*, 26(6):1341.

He, X., Lau, E. H., Wu, P., Deng, X., Wang, J., Hao, X., Lau, Y. C., Wong, J. Y., Guan, Y., Tan, X., et al. (2020). Temporal dynamics in viral shedding and transmissibility of COVID-19. *Nature medicine*, 26:672–675.

Ki, M. (2020). Epidemiologic characteristics of early cases with 2019 novel coronavirus (2019-ncov) disease in Korea. *Epidemiology and health*, 42.

Lauer, S. A., Grantz, K. H., Bi, Q., Jones, F. K., Zheng, Q., Meredith, H. R., Azman, A. S., Reich, N. G., and Lessler, J. (2020). The incubation period of coronavirus disease 2019 (COVID-19) from publicly reported confirmed cases: estimation and application. *Ann Int Med*, 172(9):577–582.

Li, Q., Guan, X., Wu, P., Wang, X., Zhou, L., Tong, Y., Ren, R., Leung, K. S., Lau, E. H., Wong, J. Y., et al. (2020). Early transmission dynamics in Wuhan, China, of novel coronavirus–infected pneumonia. *New England Journal of Medicine*.

Linton, N. M., Kobayashi, T., Yang, Y., Hayashi, K., Akhmetzhanov, A. R., Jung, S.-m., Yuan, B., Kinoshita, R., and Nishiura, H. (2020). Incubation period and other epidemiological characteristics of 2019 novel coronavirus infections with right truncation: a statistical analysis of publicly available case data. *Journal of clinical medicine*, 9(2):538.

Nishiura, H., Linton, N. M., and Akhmetzhanov, A. R. (2020). Serial interval of novel coronavirus (COVID-19) infections. *International journal of infectious diseases*.

Park, M., Cook, A. R., Lim, J. T., Sun, Y., and Dickens, B. L. (2020). A systematic review of COVID-19 epidemiology based on current evidence. *Journal of Clinical Medicine*, 9(4):967.

Rai, B., Shukla, A., and Dwivedi, L. K. (2020). Estimates of serial interval for COVID-19: A systematic review and meta-analysis. *Clinical Epidemiology and Global Health*.

Tenforde, M. W., Kim, S. S., Lindsell, C. J., Rose, E. B., Shapiro, N. I., Files, D. C., Gibbs, K. W., Erickson, H. L., Steingrub, J. S., and Smithline, H. A. (2020). Symptom duration and risk factors for delayed return to usual health among outpatients with COVID-19 in a multistate health care systems network—United States, March–June 2020. *Morbidity and Mortality Weekly Report*, 69(30):993. Publisher: Centers for Disease Control and Prevention.

Tindale, L., Coombe, M., Stockdale, J. E., Garlock, E., Lau, W. Y. V., Saraswat, M., Lee, Y.-H. B., Zhang, L., Chen, D., Wallinga, J., et al. (2020). Transmission interval estimates suggest pre-symptomatic spread of COVID-19. *MedRxiv*.

Wolfel, R., Corman, V. M., Guggemos, W., Seilmaier, M., Zange, S., Mueller, M. A., Niemeyer, D., Vollmar, P., Rothe, C., Hoelscher, M., et al. (2020). Virological assessment of hospitalized patients with covid-2019. *Nature*, 581(7809):465–469.

Wu, Z. and McGoogan, J. M. (2020). Characteristics of and important lessons from the coronavirus disease 2019 (COVID-19) outbreak in China: summary of a report of 72 314 cases from the chinese center for disease control and prevention. *JAMA*, 323(13):1239–1242.

Zhang, J., Litvinova, M., Wang, W., Wang, Y., Deng, X., Chen, X., Li, M., Zheng, W., Yi, L., Chen, X., et al. (2020). Evolving epidemiology and transmission dynamics of coronavirus disease 2019 outside Hubei Province, China: a descriptive and modelling study. *The Lancet Infectious Diseases*.

Zhou, F., Yu, T., Du, R., Fan, G., Liu, Y., Liu, Z., Xiang, J., Wang, Y., Song, B., Gu, X., et al. (2020). Clinical course and risk factors for mortality of adult inpatients with COVID-19 in Wuhan, China: a retrospective cohort study. *The Lancet*, 395(10229):1054–1062.
