# Supplementary material for: COVID-19 healthcare demand projections: Arizona
Source: PLoS One. 2020 Dec 2;15(12):e0242588. doi: 10.1371/journal.pone.0242588 (PMC7710107; doi:10.1371/journal.pone.0242588)
Supplement: Attachment — Submitted filename: ResponsetoReviewers.pdf [file pone.0242588.s003.pdf]

"COVID-19 healthcare demand projections: Arizona" Summary of Changes for the Revision #3

We thank the review team for their comments on the second revision of our paper. We have implemented the two minor changes that Reviewer 1 has indicated to be sufficient for acceptance. All of the changes are highlighted in the new version of the manuscript, and marked with the corresponding comment number.

Responses to Reviewer 1's Comments on Revision 2

Thanks to the authors' clarification. I believe the manuscript has been greatly improved and almost ready to be published. I only have two more comments.

>>> We also would like to thank the reviewer for his/her insightful comments and suggestions, which has allowed us to significantly improve the paper and its readability.

(R1.C1) *Regarding "R1.C3-discrete distribution": What I was concerned with was what data generating process the authors assumed for the observed data. From the responses, it seems that the authors did not assume any stochastic uncertainty in the relationship between the "presumed exposures" and the daily case counts and simply estimated the ODE which best describes the presumed exposures in terms of squared errors. I do not think this is considered to be the standard handling of statistical uncertainty: given the presumed "exposures," the observed data as case counts should follow some sort of counting process which involves stochastic fluctuation. I proposed the use of Poisson process to (at least partially) account for this stochastic nature of the observation. In the current implementation, the authors are essentially making an assumption that they can obtain the presumed exposure deterministically from the observed case counts. As a result, the uncertainty in the result (CI in estimates) are underevaluated. However, as the overall implication might not be largely affected, I would understand if the authors just acknowledge the limitations and possible effects of the use of deterministic process in the discussion.*

>>> We thank the reviewer for the explanation; we agree with the suggestion and added the following wording to the manuscript to reflect this point.

> ...Note that this is a relatively crude method of obtaining an "average" number of exposures on each day, ignoring the large number of uncertainties that are involved in the observations of daily case counts. As a result, we note that the prediction intervals we note below may somewhat be underestimated. In subsequent work, we have developed methods that recognize that the number of case counts resulting from a given number of exposures would be random (rather than a deterministic relationship like the one we are using here). In particular, we randomly generate exposures on randomly generated exposure dates based on observed data, but we have observed that such approaches did not make a significant impact on the performance of the initialization algorithm.

(R1.C2) *Claim of novelty: thank you for clarifying what the authors meant by "novelty." If "X-factoring" alone constitutes the novelty as you mention, Inaba (2014) is indeed irrelevant. My understanding is Inaba (2014) mathematically explains how previous incidence can be obtained from the initial condition (i.e., the current observation of cases) and thus is a generalization of the "initialization approach." Note that this paper is not intended to claim the novelty of this approach, but is just using it as a*

well-known existing concept. Regarding "X-factoring:" this approach is already familiar to infectious disease epidemiologists in a different form for an adjustment of underreporting. Often this is handled by assuming the reporting probability p and incorporate binomial reporting process as part of the model, instead of inflating the observed case counts, to appropriately account for uncertainty in reporting. I do not believe that the authors should claim the novelty of X-factoring just because it's not often used in previous studies because the method is subject to a strong assumption (negligence of stochastic fluctuation). I think the claim of novelty here is not really necessary for this paper to stand and the authors can simply drop the claim without losing its value.

>>> Done. We dropped the word "novel" from line 254 to address this comment, and changed the sentence to

> ...To account for the large rate of undetected infections, we have devised an intuitive approach using an X-factor initialization scheme where we multiply the number of eventually detected-exposed individuals by the X-factor to estimate the underlying overall exposures on a given presumed exposure day.

If these two points have been addressed, I am happy for this paper to be published.
>>> We thank the reviewer again for his/her comments and contributions that improved the paper.
